# Endogenous analgesia mediated by CD4^+^ T lymphocytes is dependent on enkephalins in mice

**DOI:** 10.1186/s12974-016-0591-x

**Published:** 2016-06-01

**Authors:** Lilian Basso, Jérôme Boué, Karim Mahiddine, Catherine Blanpied, Sébastien Robiou-du-Pont, Nathalie Vergnolle, Céline Deraison, Gilles Dietrich

**Affiliations:** IRSD, Université de Toulouse, INSERM, INRA, ENVT, UPS, Toulouse, France; CPTP, Université de Toulouse, CNRS, INSERM, UPS, Toulouse, France

**Keywords:** T lymphocytes, Enkephalin, β-endorphin, Inflammation, Pain

## Abstract

**Background:**

T cell-derived opioids play a key role in the control of inflammatory pain. However, the nature of opioids produced by T cells is still matter of debate in mice. Whereas β-endorphin has been found in T lymphocytes by using antibody-based methods, messenger RNA (mRNA) quantification shows mainly mRNA encoding for enkephalins. The objective of the study is to elucidate the nature of T cell-derived opioids responsible for analgesia and clarify discrepancy of the results at the protein and genetic levels.

**Methods:**

CD4^+^ T lymphocytes were isolated from wild-type and enkephalin-deficient mice. mRNA encoding for β-endorphin and enkephalin was quantified by RT-qPCR. The binding of commercially available polyclonal anti-endorphin antibodies to lymphocytes from wild-type or enkephalin knockout mice was assessed by cytofluorometry. Opioid-mediated analgesic properties of T lymphocytes from wild-type and enkephalin-deficient mice were compared in a model of inflammation-induced somatic pain by measuring sensitivity to mechanical stimuli using calibrated von Frey filaments.

**Results:**

CD4^+^ T lymphocytes expressed high level of mRNA encoding for enkephalins but not for β-endorphin in mice. Anti-β-endorphin polyclonal IgG antibodies are specific for β-endorphin but cross-react with enkephalins. Anti-β-endorphin polyclonal antibodies bound to wild-type but not enkephalin-deficient CD4^+^ T lymphocytes. Endogenous regulation of inflammatory pain by wild-type T lymphocytes was completely abolished when T lymphocytes were deficient in enkephalins. Pain behavior of immune-deficient (i.e., without B and T lymphocytes) mice was superimposable to that of mice transferred with enkephalin-deficient lymphocytes.

**Conclusions:**

Rabbit polyclonal anti-β-endorphin serum IgG bind to CD4^+^ T lymphocytes because of their cross-reactivity towards enkephalins. Thus, staining of T lymphocytes by anti-β-endorphin polyclonal IgG reported in most of studies in mice is because of their binding to enkephalins. In mice, CD4^+^ T lymphocytes completely lose their analgesic opioid-mediated activity when lacking enkephalins.

## Background

**Fig. 1:**
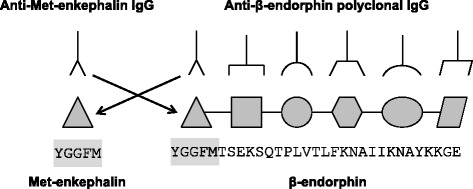
Schematic representation of the potential cross-reactivity of anti-Met-enkephalin and anti-β-endorphin polyclonal IgG antibodies. The amino acid sequence of Met-enkephalin (five amino acids corresponding to only one epitope, *gray box*) is identical to the first five amino acids of the β-endorphin and may be recognized by immune serum IgG specifically raised against Met-enkephalin () or β-endorphin ()

In inflammatory conditions, pain is regulated by endogenous opioids produced by immune cells. Inflammation-induced somatic pain is first attenuated by opioid-producing inflammatory cells including neutrophils and macrophages, and then, few days later, abolished by opioid-producing T lymphocytes entering the site of inflammation [1–5]. Although all three μ, δ, and k subclasses of opioid receptors are expressed in peripheral endings of afferent neurons, only the δ-type opioid receptor (DOR) is involved in the spontaneous regulation of somatic complete Freund’s adjuvant (CFA)-induced inflammatory pain in mice [6–9]. In line with the pivotal role of DOR in the endogenous regulation of inflammatory somatic pain in mice, we reported that effector T lymphocytes produce enkephalins, the most selective endogenous ligands for DOR, but virtually not β-endorphin [1, 6, 10]. Although enkephalins are commonly described in mouse T lymphocytes, numerous laboratories also found β-endorphin. All the studies reporting β-endorphin in mouse T lymphocytes were performed by using immunochemistry methods with rabbit anti-β-endorphin polyclonal IgG antibodies [11–16]. The specificity of anti-β-endorphin IgG antibodies towards β-endorphin was clearly demonstrated by the absence of cross-species binding of the Ig heavy chain constant region to the Fc receptors expressed on leukocytes (staining with isotype-matched control antibodies) together with the complete inhibition of the anti-β-endorphin IgG binding by β-endorphin. However, these experiments that showed the specificity of polyclonal anti-β-endorphin IgG towards the multiple epitopes of β-endorphin polypeptide, did not exclude that they could cross-react (*i.e.*, to recognize the same epitope on two distinct antigens) with Met-enkephalin, a five-amino-acid peptide corresponding to one epitope of the β-endorphin (Fig. 1). Given that messenger RNA (mRNA) expression analysis showed that activated mouse T lymphocytes expressed mRNA encoding for proenkephalin (PENK) but not for proopiomelanocortin (POMC) [1, 6, 10], we assumed that the binding of anti-β-endorphin IgG antibodies to T lymphocytes was due to the recognition of Met-enkephalin.

Here, we show that the intracytoplasmic staining of activated mouse T lymphocytes by anti-β-endorphin polyclonal IgG is due to cross-reactivity towards enkephalin. Moreover, we show that the endogenous regulation of inflammatory somatic pain by CD4^+^ T lymphocytes in mice is completely abrogated when T lymphocytes are deficient in enkephalins.

## Methods

### Animals

C57BL/6 mice were provided from Janvier (Le Genest Saint Isle, France) and recombination-activating gene 2-deficient C57BL/6 (RAG2^−/−^) mice were from ANEXPLO platforms (UMS 006, Toulouse, France). Pre-proenkephalin knockout (PENK^−/−^) mice were the B6.129-*Penk*-rs^tm1Pig^/J strain with a genetic background C57BL/6 (MHC H-2 Haplotype b) provided by The Jackson Laboratory (Bar Harbor, Maine, USA). All mice used in the study were 8–10-week-old male weighing 20–25 g. Mice were housed at a temperature between 20 and 22 °C and maintained under a 12-h light/dark cycle in sawdust coated transparent cages. Animals were housed by three or two in ventilated cages with chow and water ad libitum. All experiments involving animals were performed in accord with ethical guidelines (INSERM) and were approved by the Midi-Pyrénées (France) ethics committee (application Number MP/06/73/10/12).

### Isolation and activation of CD4^+^ T lymphocytes

CD4^+^ T lymphocytes were isolated from splenocytes using cell negative isolation kits according to the manufacturer’s instructions (Invitrogen Dynal AS, Oslo, Norway). Twenty-four-well cell culture plates (Corning, Life Sciences, Amsterdam, Netherlands) previously coated with 2.5 μg mL^−1^ of anti-CD3 (clone 145-2C11) and 2.5 μg mL^−1^ of anti-CD28 (clone 37.51) monoclonal antibodies (mAbs) (BD Biosciences, San Jose, CA) were seeded with 5 × 10^5^ purified naive CD4^+^ T cells (more than 92 % pure) in RPMI-1640 medium (GIBCO Life Technologies, Paisley, UK) supplemented with 10 % heat inactivated fetal calf serum (GIBCO Life Technologies), 1 % non-essential amino acids, 4-mM l-glutamine, 1-mM sodium pyruvate, 100-IU/ml penicillin, 100-μg/ml streptomycin (GIBCO-BRL), 10-mM HEPES (4-(2-hydroxyethyl)-1-piperazineethanesulfonic acid), and 2 × 10^-5^ M 2-β-Mercapto-ethanol.

### Cytofluorometric analysis of antibody activity

10^6^ cells were incubated with anti-CD16/CD32 (mouse Fc block™, clone 2.4G2, BD Biosciences), fixed and permeabilized with BD Cytofix/Cytoperm solution before being incubated with 100 μL of either rabbit anti-β-endorphin polyclonal IgG (10 μg mL^−1^, Merck-Millipore (Chemicon International), Temecula, CA), rabbit anti-Met-enkephalin polyclonal IgG (10 μg mL^−1^, Merck-Millipore (Chemicon International), Temecula, CA), or rabbit non-immune serum IgG (10 μg mL^−1^, Jackson Immunoresearch Lab) diluted in PBS containing 1 % fetal calf serum and 2 mM EDTA. After washing, cell-bound antibodies were revealed using fluorescein isothiocyanate (FITC)-conjugated goat anti-rabbit IgG secondary antibodies (BD Biosciences). Data were collected on 20,000 cells by forward and side scatter intensity on an FACs calibur flow cytometer (Becton Dickinson, Franklin Lakes, NJ) and were subsequently analyzed using the Flow Jo software (Tree Star Inc., Ashland, OR). The neuroepithelioma cell line SK-N-MC was grown in DMEM supplemented with 10 % fetal-calf serum at 37 °C with 5 % CO_2_ in air atmosphere until the cells reach 80 % confluence [17].

For inhibition experiments, rabbit anti-β-endorphin or anti-Met-enkephalin antibodies were incubated with 10 μM of soluble β-endorphin (Sigma-Aldrich, St. Louis, MO) or Met-enkephalin (Sigma-Aldrich, St. Louis, MO) for 20 min at 37 °C before being added to the cells overnight at 4 °C [18]. After washing, cell-bound antibodies were revealed using fluorescein isothiocyanate (FITC)-conjugated goat anti-rabbit IgG secondary antibodies as described above.

### Real-time PCR analysis

Total RNA was isolated by TRIzol™ Reagent and reverse-transcribed with Moloney murine leukemia virus reverse transcriptase using random hexamers for priming. Transcripts encoding hypoxanthine phosphoribosyl transferase (HPRT), proenkephalin (PENK), and proopiomelanocortin (POMC) were quantified by real-time PCR in mouse CD4^+^ T cells. Forward and reverse primers were 5′-GTTCTTTGCTGACCTGCTGGAT-3′ and 5′-CCCCGTTGACTGATCATTACAG-3′ for HPRT, 5′-CGACATCAATTTCCTGGCGT-3′ and 5′-AGATCCTTGCAGGTCTCCCA-3′ for PENK, and 5′-TGGCCCTCCTGCTTCAGAC-3′ and 5′-CAGCGAGAGGTCGAGTTTGC-3′ for POMC, [19]. The target gene expression was normalized to the HPRT mRNA and quantified relative to a standard complementary DNA (cDNA) (calibrator sample) prepared from mouse brain using the 2^−ΔΔ*C*T^ method, where ΔΔ*C*_T_ = Δ*C*_T sample_ − Δ*C*_T calibrator_ [20]_._

### Spleen cell transfer, immunization, and measurement of somatic nociception in mice

Splenocytes (30 × 10^6^) isolated from either wild-type C57BL/6 (PENK^+/+^) or pre-proenkephalin knockout (PENK^−/−^) mice were intravenously (i.v.) injected into immunodeficient RAG_2_^−/−^ mice. Mice were housed by three or two in ventilated cages. The next day, recipient mice were immunized by injecting subcutaneously (s.c.) into hind footpads ovalbumin (OVA, Sigma Chemical Co., St Louis, MO) emulsified in complete Freund’s adjuvant (CFA) (50 μl at 1 mg mL^−1^).

Mechanical withdrawal thresholds were measured using ascending series calibrated von Frey filaments of binding forces ranging from 0.04 to 2 g (Stoelting, Wood Dale, IL, USA), applied onto the plantar surface of mice. Threshold to mechanical stimuli was calculated as the force value of the von Frey filament triggering three paw withdrawals over five applications. Responses to mechanical stimuli were recorded before, and daily after immunization. The groups of mice treated with naloxone methiodide (NLX) were injected (10 μl at 2 mg mL^−1^) into the ankle of the inflamed hind paw, 30 min before each pain assessment [1]. Naloxone methiodide treatment was initiated on day 3 after immunization when T cells become the predominant immune cells at the inflammatory site [2].

### Statistical analysis

Data are expressed as mean± SEM. Due to the skewed distribution of the responses, non-parametric tests were applied on longitudinal data using a ranked-based approach on factorial experiments [21, 22]. Statistics tests were computed using modified ANOVAs with the R software package “nparLD” for longitudinal data [23]. Mann-Whitney-Wilcoxon tests were used for the post hoc analysis using R 3.0.2. *p* < 0.05 was considered as significant.

The statistical model assessed response over time between treatment groups including an interaction term between treatment groups and days of measurement as fixed effects and a random effect to account for the mice repeated measures over time. The variable treatment was considered as factorial experiment. We first tested whether response over time differed across the five treatment groups (RAG2^−/−^, PENK^+/+^, PENK^+/+^ NLX, PENK^−/−^ and PENK^−/−^ NLX). Significant (*p* < 0.05) differences of response between groups allowed performing subsequent analysis of a random effect to account for the mice repeated measures over time. All pairwise combinations of treatment were tested to assess their effect on response. Finally, we also compared response between treatments at each time point using a Mann-Whitney-Wilcoxon test.

## Results

### Anti-β-endorphin polyclonal IgG antibodies cross-react with enkephalins expressed in activated T lymphocytes

**Fig. 2:**
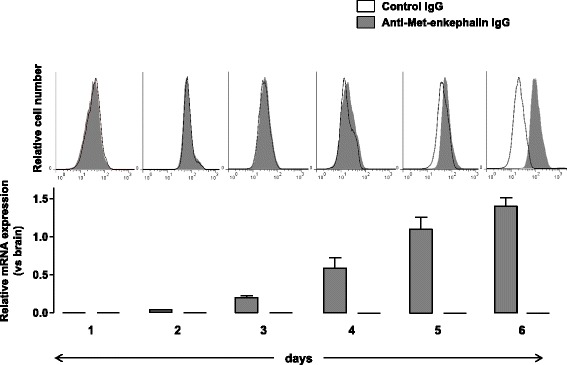
Proenkephalin is upregulated upon T cell activation. Naive CD4^+^ T lymphocytes isolated from wild-type mice were stimulated with both anti-CD3 and anti-CD28 mAbs for 6 days. Expression levels of mRNA encoding for PENK (*gray histogram*) and POMC (*white histogram*) were quantified by real-time PCR in CD4^+^ T lymphocytes from day 1 to day 6 (*lower panels*). mRNA content was normalized to the HPRT mRNA and quantified relative to standard mouse brain cDNA. Gene expression was assessed in at least five independent experiments run in duplicate. Results (mean ± SEM) are expressed relative to PENK or POMC mRNA expression in the mouse brain. Intracytoplasmic accumulation of Met-enkephalin-containing peptides was then assessed by cytofluorometry (*upper panels*). CD4^+^ T lymphocytes recovered each day of the stimulation were incubated with either control rabbit IgG (*white histogram*) or rabbit anti-Met-enkephalin IgG antibodies (*gray histogram*). The figure shows one representative experiment out of three performed

Purified naive wild-type CD4^+^ T lymphocytes were stimulated with a cocktail of anti-CD3 and anti-CD28 antibodies for 6 days in vitro. Expression of mRNA encoding for PENK and POMC was then quantified by real-time PCR. As shown in Fig. 2, mRNA encoding PENK was upregulated upon activation of CD4^+^ T lymphocytes. By contrast, mRNA encoding POMC remained undetectable on day 6 of activation (Fig. 2, lower panel). The upregulation of PENK mRNA was correlated with an intracytoplasmic accumulation of Met-enkephalin-containing peptides as assessed by cytofluorometry (Fig. 2, upper panel).

**Fig. 3:**
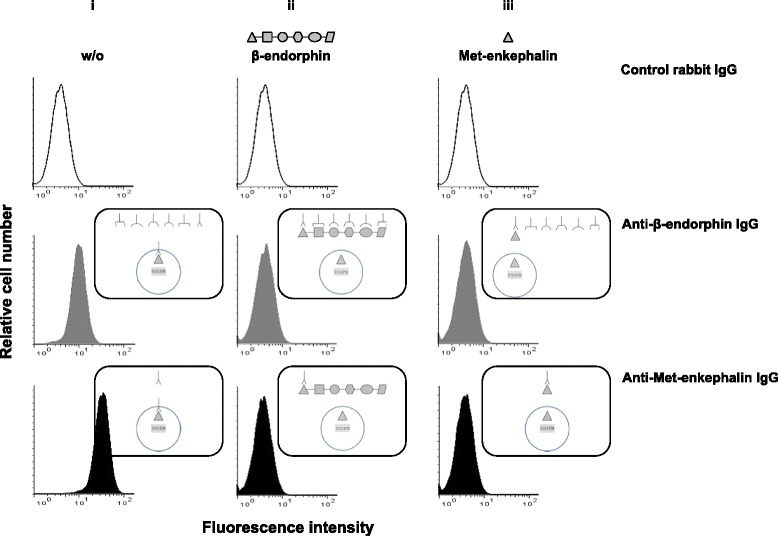
Anti-β-endorphin polyclonal IgG antibodies recognize enkephalins expressed in activated T lymphocytes as assessed by cytofluorometry. CD4^+^ T lymphocytes isolated from wild-type C57Bl/6 PENK^+/+^ mice were stimulated with both anti-CD3 and anti-CD28 mAbs for 6 days and intracellularly stained with control rabbit non-immune serum IgG (*upper panels*), rabbit anti-β-endorphin polyclonal IgG antibodies (*middle panels*), or rabbit anti-Met-enkephalin polyclonal IgG antibodies (*lower panels*). The figure depicts the binding of each of the three rabbit polyclonal IgG in the absence (i) or in the presence of an excess of soluble β-endorphin (ii) or Met-enkephalin (iii). The figure shows one representative experiment out of four performed. Schematic interpretation of the experiments is shown on the right of each histogram

Contrasting with our results at the genetic level, a number of studies found β-endorphin in mouse T lymphocytes by using anti-β-endorphin polyclonal serum IgG. Given that Met-enkephalin matches to the NH_2_ terminal segment of the β-endorphin sequence, we assessed the ability of anti-β-endorphin polyclonal IgG to cross-react with Met-enkephalin (Fig. 1). As shown in Fig. 3, polyclonal rabbit IgG directed against β-endorphin as well as those directed against Met-enkephalin bound to activated T lymphocytes, while control rabbit non-immune serum IgG did not. The binding of anti-β-endorphin and anti-enkephalin IgG to T lymphocytes was wholly inhibited when they were pre-incubated with either soluble β-endorphin or Met-enkephalin (Fig. 3, middle and right panels) indicating that anti-β-endorphin and anti-enkephalin IgG recognize both β-endorphin and enkephalin. The complete inhibition by soluble Met-enkephalin of the binding of anti-β-endorphin IgG to T lymphocytes (Fig. 3, right panel) demonstrates that anti-β-endorphin polyclonal IgG recognize Met-enkephalin but not β-endorphin in T lymphocytes.

**Fig. 4:**
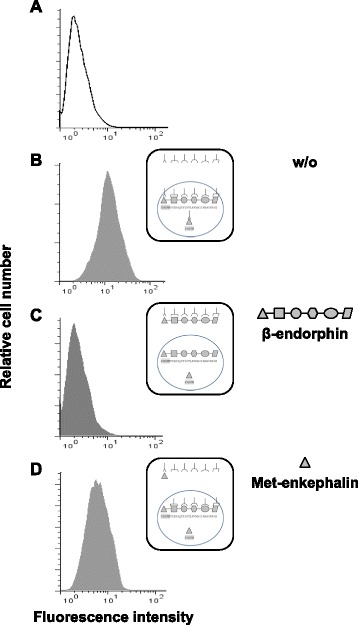
The binding of anti-β-endorphin antibody to β-endorphin-expressing SK-N-MC cells is partially inhibited by soluble Met-enkephalin as assessed by cytofluorometry. The human neuroblastoma cell line SK-N-MC expressing both enkephalin and β-endorphin was intracellularly stained with control rabbit non-immune serum IgG (**a**) or rabbit anti-β-endorphin polyclonal IgG antibodies in the absence (**b**) or in the presence of an excess of soluble β-endorphin (**c**) or Met-enkephalin (**d**). The figure shows one representative experiment out of three performed. Schematic interpretation of the experiments is shown on the right of each histogram

By contrast, the binding of polyclonal anti-β-endorphin IgG to SK-N-MC cells, which express low levels of POMC mRNA (data not shown), was totally inhibited by β-endorphin (Fig. 4c) and partially by Met-enkephalin (Fig. 4d), demonstrating that the pre-absorption of anti-β-endorphin IgG antibodies with Met-enkephalin does not neutralize the binding to the other epitopes of the β-endorphin (Fig. 4d).

**Fig. 5:**
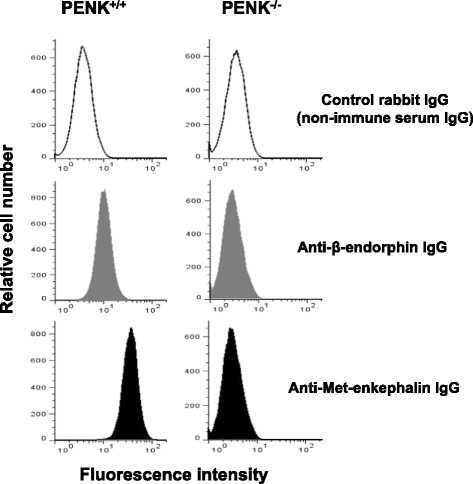
Anti-β-endorphin polyclonal IgG antibodies do not bind to activated lymphocytes from pre-proenkephalin knockout mice as assessed by cytofluorometry. CD4^+^ T lymphocytes isolated from wild-type C57Bl/6 (PENK^+/+^) mice (*left panels*) or pre-proenkephalin knockout (PENK^−/−^) mice (*right panels*) were stimulated with both anti-CD3 and anti-CD28 mAbs for 6 days and intracellularly stained with either control rabbit non-immune serum IgG (*upper panels*), rabbit anti-β-endorphin polyclonal IgG antibodies (*middle panels*), or rabbit anti-Met-enkephalin polyclonal IgG antibodies (*lower panels*). The figure shows one representative experiment out of three performed

In order to confirm that the binding of polyclonal anti-β-endorphin antibodies to activated mouse CD4^+^ T lymphocytes was due to the recognition of Met-enkephalin, we assessed the binding of anti-β-endorphin antibodies to enkephalin-deficient CD4^+^ T lymphocytes. As shown in Fig. 5, anti-β-endorphin antibodies bound to wild-type but not to PENK-deficient CD4^+^ T lymphocytes.

Taken together, the data show that (1) activated CD4^+^ T lymphocytes do not express β-endorphin in mice and (2) the binding of rabbit polyclonal anti-β-endorphin serum IgG to activated mouse CD4^+^ T lymphocytes is because of their cross-reactivity towards enkephalins.

### Analgesic activity of activated CD4^+^ T lymphocytes is dependent on enkephalins

**Fig. 6:**
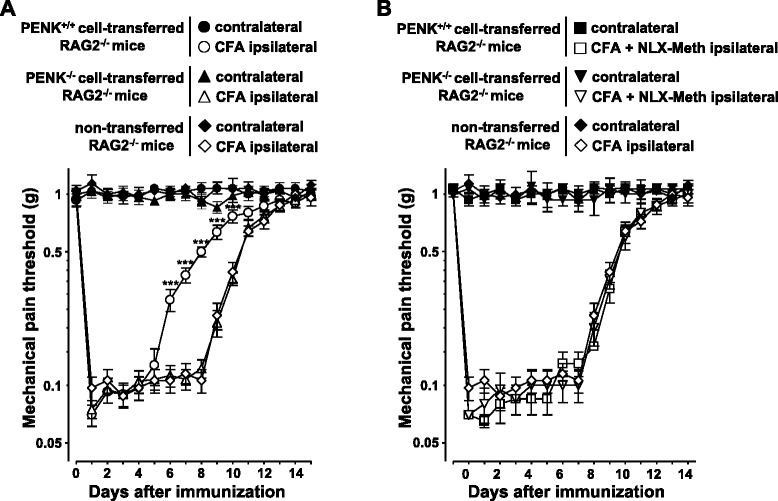
Anti-nociceptive T cell activity is mediated by enkephalins in mice. **a** Splenocytes from PENK^+/+^ wild-type C57Bl/6 (*circle*) or PENK^−/−^ pre-proenkephalin knockout (*triangle*) mice were transferred i.v. into immune-deficient RAG-2^−/−^ mice (*n* = 12 for each group of mice). The next day, mice were immunized s.c. into hind footpads with OVA in CFA. Immunized non-transferred RAG2^−/−^ mice (*n* = 10, *diamond*) were used as control. Inflammatory pain was monitored by measuring sensitivity of the mice to mechanical stimuli using the von Frey test. Data are expressed as mean ± SEM paw withdrawal thresholds measured in inflamed ipsilateral immunized paws (*open symbol*) and contralateral non-injected paws (*closed symbol*). Results were obtained from two sets of experiments performed on groups of six (T cell transferred) or five (RAG2^−/−^) mice. ***p < 0.001 (comparison with immunized non-transferred RAG-2^−/−^ mice). **b** From day 3 until the end of the experiment, recipient RAG2^−/−^ mice transferred with either PENK^+/+^ (*square*; *n* = 6) or PENK^−/−^ (*inverted triangle*; *n* = 6) splenocytes were daily injected into inflamed hind paw with 10 μL of naloxone methiodide (NLX-Meth) at 2 mg mL^−1^, 30 min before pain assessment. Mice were randomly assigned into each group in blind experiments in which the treatment and pain assessment were performed by two different experimenters. Data are expressed as mean ± SEM paw withdrawal thresholds measured in inflamed ipsilateral immunized paws (*open symbol*) and contralateral non-injected paws (*closed symbol*). Results were obtained from two sets of experiments performed on groups of mice mice

The fundamental role of T cell-derived enkephalins in the regulation of inflammatory pain was appreciated by comparing mechanical sensitivity of mice in which lymphocytes express or not enkephalins. Immune-deficient RAG2^−/−^ mice transferred with splenocytes originating from either wild-type C57BL/6 (PENK^+/+^) or enkephalin-deficient knockout (PENK^−/−^) mice were injected with complete Freund’s adjuvant (CFA). The nociceptive response to mechanical stimuli was then estimated by using calibrated von Frey filaments. Basal mechanical sensitivity measured in contralateral hind paw (non-injected with CFA) was superimposable in all the groups of mice (Fig. 6). Within 5 days following the injection of CFA, mechanical sensitivity of the ipsilateral paw was similar in RAG2^−/−^ mice transferred with PENK^+/+^ or PENK^−/−^ lymphocytes. The sensitivity to mechanical stimuli decreased in RAG2^−/−^ transferred with PENK-expressing T lymphocytes 3 days before those transferred with PENK-deficient T lymphocytes (Fig. 6a). From day 6 to day 10, the nociceptive response to mechanical stimuli was significantly lower in mice transferred with wild-type PENK^+/+^ T lymphocytes as compared to those transferred with PENK-deficient T lymphocytes (*F* = 23, *p* < 0.00001), (Fig. 6a). Mechanical sensitivity was similar between non-transferred immunodeficient RAG2^−/−^ mice that do not have lymphocytes and those transferred with enkephalin-deficient lymphocytes (Fig. 6a) (*F* = 0.13, *p* = 0.72) indicating that enkephalins are the main opioids released by T lymphocytes within the inflammatory site at the late stage of CFA-induced inflammation. The fundamental role of enkephalins in T cell-mediated analgesia was confirmed by using naloxone methiodide, an antagonist of the three classes of opioid receptors unable to cross the blood-brain barrier (Fig. 6b). Whereas local administration of naloxone methiodide worsened CFA-induced inflammatory pain in mice with enkephalin-producing T lymphocytes (*F* = 21, *p* = 0.00001), it had no effect in mice with enkephalin-deficient T lymphocytes (Fig. 6) (*F* = 0.44, *p* = 0.51), (Fig. 6).

## Discussion

During microbe-induced inflammation, leukocytes at the inflammatory site produce opioids and contribute to modulate the intensity of the inflammation-related pain [24]. As shown in CFA-induced pain model in mice, the contribution of leukocytes in regulating pain varies at the different steps of the inflammatory process. At the early stage of inflammation, neutrophils and monocytes that first enter the site of inflammation produce opioids but the release of their opioid content is insufficient to spontaneously abolish painful perception [3, 19, 25]. From the third day of inflammation, effector CD4^+^ T lymphocytes generated in response to bacterial antigens contained in CFA migrate into the inflammatory site to enhance innate immune response against microbes. Effector CD4^+^ T lymphocytes, which become the predominant immune cell subset in the inflammatory site, release opioids. Activation of opioid receptors expressed on sensory neurons innervating the inflamed area by T lymphocyte-derived opioids is, at this time, sufficiently efficient to relieve from CFA-induced inflammatory pain [1, 2]. The ability of CD4^+^ T lymphocytes to produce opioids is acquired upon antigen priming in draining lymph nodes [1] and correlates with the stimulatory potency of antigen-presenting cells [6]. Effector CD4^+^ T lymphocytes entering the inflammatory site release their opioid content upon stimulation by the cognate antigens in situ [6]. A 6-day latency between immunization (*i.e*., CFA injection) and analgesia (Fig. 6) is required for antigen-specific priming, clonal expansion, and differentiation of CD4^+^ T lymphocytes within the draining lymph nodes and their recruitment in sufficient number at the site of inflammation [1, 26, 27]. The kinetics of the allodynic response to mechanical stimuli of immunodeficient mice transferred with PENK^+/+^ T lymphocytes was similar to that of wild-type immunocompetent mice [1, 8]. The production of enkephalins by effector T lymphocytes generated in response to CFA (mycobacterium tuberculosis) reduces almost by two the duration of somatic inflammatory pain in mice [1, 8, 26]. Given the anti-inflammatory properties of endogenous opioids [28–31], the analgesic effects of T cell-derived enkephalins in the chronic model of CFA-induced inflammation are certainly not restricted to their inhibitory action on sensory neurons [10].

The production of endogenous opioids by effector CD4^+^ T lymphocytes in humans and rodents is commonly admitted, but the nature of the opioid peptides may differ across animal species [1, 4, 32, 33]. Contrasting with the experiments performed in humans and rats, which described at both genetic and protein levels [34–36] the expression of β-endorphin in immune cells including T lymphocytes, the studies performed in mice only used antibody-based methods. To our knowledge, all the studies describing β-endorphin in mouse T lymphocytes have been performed by immunochemistry methods using polyclonal anti-β-endorphin antibodies [11–16]. Accordingly, Met-enkephalin, which constitutes the first five amino acids of the NH_2_ terminus of the β-endorphin, may be recognized as an epitope by immune serum IgG raised against β-endorphin. Polyclonal anti-β-endorphin antibodies are specific for β-endorphin but cross-react with Met-enkephalin, and thereby, they bind to cells producing β-endorphin and/or Met-enkephalin. Their binding is always fully inhibited by soluble β-endorphin even if the cells only produce Met-enkephalin. However, the total inhibition of the anti-β-endorphin IgG binding by soluble Met-enkephalin indicates that the target cells only express Met-enkephalin (*i.e*., the anti-β-endorphin antibody staining is due to the recognition of Met-enkephalin). Thus, the absence of β-endorphin-encoding mRNA (POMC) in T lymphocytes [1, 6, 10], the full inhibition of the anti-β-endorphin antibody binding to wild-type CD4^+^ T lymphocytes by Met-enkephalin, and the inability of anti-beta-endorphin antibodies to bind to PENK^−/−^ lymphocytes argue for the expression of enkephalins but not β-endorphin in mouse T lymphocytes.

All the three subclasses of opioid receptors are expressed on sensory neurons innervating skin and gastrointestinal tract [37, 38], and CD4^+^ T lymphocytes have been shown to be the most effective regulators of pain upon chronic inflammation including intestinal inflammation in mice [1, 6, 10, 13–15]. However, although opioid drugs specific for each of the three opioid receptors are efficient to relieve inflammatory pain when administered in periphery [6, 39–41], the molecular nature of the endogenous opioids produced by CD4^+^ T lymphocytes remains subject to controversy in mice [42]. Because of the structural similarities between endogenous opioid neuropeptides, and thereby the poor reliability of anti-opioid immune sera, we used a genetic approach to show the pivotal role of enkephalins in the analgesic property of CD4^+^ T lymphocytes. We show, in CFA-induced pain model in mice, that CD4^+^ T lymphocytes lacking enkephalins completely lose their analgesic activity. Furthermore, the mechanical sensitivity of mice with enkephalin-deficient lymphocytes was superimposable to that of mice without lymphocytes indicating that the absence of enkephalins in T lymphocytes was not compensated by another opioid.

## Conclusions

Endogenous regulation of CFA-induced inflammatory pain by CD4^+^ T lymphocytes is primarily mediated by enkephalins in mice suggesting that β-endorphin would be more relevant in rats and humans.

## Abbreviations

CFA, complete Freund’s adjuvant; DOR, delta-type opioid receptor; HPRT, hypoxanthine phosphoribosyl transferase; mAb, monoclonal antibody; NLX, naloxone methiodide; OVA, ovalbumin; PENK, proenkephalin; POMC, proopiomelanocortin; RAG2^−/−^, recombination-activating gene 2-deficient
